# A severe case of Israeli spotted fever with pleural effusion in Italy

**DOI:** 10.1007/s15010-021-01693-8

**Published:** 2021-09-09

**Authors:** Cristoforo Guccione, Claudia Colomba, Raffaella Rubino, Celestino Bonura, Antonio Anastasia, Stefano Agrenzano, Valentina Caputo, Giovanni Maurizio Giammanco, Antonio Cascio

**Affiliations:** 1 Department of Health Promotion, Mother and Child Care, Internal Medicine and Medical Specialties, University of Palermo, 90127 Palermo, Italy; 2 Infectious and Tropical Disease Unit, AOU Policlinico “P. Giaccone”, Via del Vespro 129, 90127 Palermo, Italy

**Keywords:** Mediterranean spotted fever, Pleural effusion, *Rickettsiales*, *Rickettsia*, *Rickettsiosis*, Italy

## Abstract

**Background:**

The most common Italian rickettsiosis is Mediterranean Spotted Fever (MSF). MSF is commonly associated with a symptom triad consisting of fever, cutaneous rash, and inoculation eschar. The rash is usually maculopapular but, especially in severe presentations, may be petechial. Other typical findings are arthromyalgia and headache. Herein, we describe for the first time an unusual case of Israeli spotted fever (ISF) associated with interstitial pneumonia and pleural effusion in which *R. conorii *subsp*. israelensis* was identified by molecular methods in the blood, as well as in the pleural fluid.

**Case presentation:**

A 72-year-old male presented with a 10-day history of remittent fever. On admission, the patient’s general condition appeared poor with confusion and drowsiness; the first assessment revealed a temperature of 38.7°, blood pressure of 110/70 mmHg, a blood oxygen saturation level of 80% with rapid, frequent, and superficial breathing using accessory muscles (28 breaths per minute), and an arrhythmia with a heart rate of 90 beats per minute. qSOFA score was 3/3. Chest CT revealed ground-glass pneumonia with massive pleural effusion. Petechial exanthema was present diffusely, including on the palms and soles, and a very little eschar surrounded by a violaceous halo was noted on the dorsum of the right foot. Awaiting the results of blood cultures, broad-spectrum antibiotic therapy with meropenem 1 g q8h, ciprofloxacin 400 mg q12h, and doxycycline 100 mg q12h was initiated. Doxycycline was included in the therapy because of the presence of petechial rash and fever, making us consider a diagnosis of rickettsiosis. This suspicion was confirmed by the positivity of polymerase chain reaction on whole blood for *R. conorii *subsp*. israelensis.* Thoracentesis was performed to improve alveolar ventilation. *R. conorii *subsp*. israelensis* was again identified in the pleural fluid by PCR technique. On day 4 the clinical condition worsened. Blood exams showed values suggestive of secondary hemophagocytic lymphohistiocytosis; 4 out of 8 diagnostic criteria were present and empirical treatment with prednisone was started resulting in a gradual improvement in general condition.

**Conclusions:**

Israeli spotted fever may be a severe disease. A high index of suspicion is required to promptly start life-saving therapy. Pleural effusion and interstitial pneumonia may be part of the clinical picture of severe *rickettsial* disease and should not lead the physician away from this diagnosis.

## Introduction

The most common Italian rickettsiosis is Mediterranean spotted fever (MSF). About 400 cases of MSF are reported every year, most of which in people residing in Sicily, Sardinia and Southern Italy with a mortality of < 3% [1, 2]*.* MSF is commonly associated with a symptom triad consisting of fever, cutaneous rash, and tache noire*.* The rash is usually maculopapular but, especially in severe presentations, can be petechial. The tache noire, translatable from French as “black spot”, is the typical, painless and non-pruritic eschar at the site of the arthropod bite. Other typical findings are arthromyalgia and headache. However, in recent years, other rickettsiosis such as TIBOLA/DEBONEL/SENLAT (Tick-Borne Lymphadenopathy/Dermacentor Borne Necrosis Erythema and Lymphadenopathy /Scalp Eschar and Neck Lymphadenopathy After Tick Bite), and many other *Rickettsia* spp. or subspecies have been identified in humans, vector arthropods and animals [3].

Here, we describe an unusual case of Israeli spotted fever (ISF) associated with interstitial pneumonia and pleural effusion in which *R. conorii *subsp*. israelensis* was identified by molecular methods in the blood, as well as in the pleural fluid.

## Case report

**Fig. 1:**
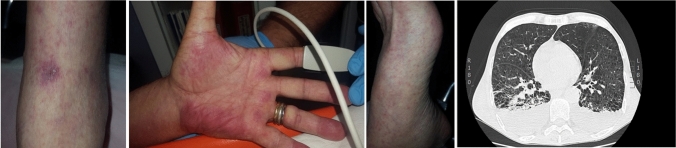
Little eschar surrounded by a violaceous halo (tache noire), rash involving palms and feet, and computer tomography showing interstitial pneumonia and massive pleural effusion

A 72-year-old male smoker with no history of major illness presented with a ten-day history of remittent fever. He was previously admitted to a primary hospital in another district from which he was discharged with home therapy consisting of ceftriaxone 1 g qd and steroids. The home therapy did not improve his condition and, for this reason, on 4 August 2018, he was admitted to our hospital. On admission, the patient’s general condition appeared poor with confusion and drowsiness, he was also feverish and had generalized edema; the first assessment revealed a temperature of 38.7°, blood pressure of 110/70 mmHg, a blood oxygen saturation level of 80% with rapid, frequent and superficial breathing using accessory muscles (28 breaths per minute), and an arrhythmia with a heart rate of 90 beats per minute. qSOFA score was 3/3. Chest CT revealed ground-glass pneumonia with massive pleural effusion. Petechial exanthema was present diffusely, including on the palms and soles, and a very little eschar surrounded by a violaceous halo was noted on the dorsum of the right foot (Fig. 1). Awaiting the results of blood cultures, broad-spectrum antibiotic therapy with meropenem 1 g q8h, ciprofloxacin 400 mg q12h, and doxycycline 100 mg q12h was initiated. Doxycycline was included in the therapy because of the presence of petechial rash and fever, making us consider a diagnosis of rickettsiosis. This suspicion was confirmed by the positivity of polymerase chain reaction on whole blood for *R. conorii *subsp*. israelensis.* Rickettsial DNA was detected from full blood specimens with a highly sensitive real-time PCR assay for the detection of spotted fever and typhus group *rickettsiae* using previously published primers and the probe to the *Rickettsia rickettsii* citrate synthase gene, gltA [4]. The CSisr-P probe (5′-FAM-TGT AAT AGC AAG AAT CGT AGG CTG GAT G-TAMRA-3′) was specifically designed from a highly conserved region of the citrate synthase gene to detect *R. conorii *subsp*. israelensis* in addition to SFG *rickettsiae*. To improve alveolar ventilation a thoracentesis was performed with clinical improvement. The fluid was sent to the laboratory for microbiological analysis and *R. conorii *subsp*. israelensis* was again identified with the PCR technique. Tests to detect specific antibodies to influenza A and B viruses, parainfluenza viruses, adenoviruses, respiratory syncytial virus, *Mycoplasma pneumoniae*, *Chlamydia* spp., and *Legionella pneumophila* were all negative. On day 4, the clinical condition worsened. Blood exams showed the following results: fibrinogen (83 mg/dl), antithrombin III (39%), prolonged aPTT (37 s) and anemia (Hb 7.8 g/dl). These values and the infective trigger appeared suggestive of secondary hemophagocytic lymphohistiocytosis (sHLH) [5]. In fact, 4 out of 8 diagnostic criteria were present (fever, cytopenia of 2 or more cellular lineages in peripheral blood, fibrinogenemia lower than 150 mg/dl, ferritinemia higher than 500 ng/dl). Notwithstanding, the patient did not consent for bone marrow biopsy. Hence, the suspicion led us to start empirical treatment with prednisone (25 mg twice a day); gradual improvement of general condition and hematic values was observed in the following days. After seven days, the antibiotic therapy was discontinued and on the 10th day from hospital admission the patient was discharged.

## Discussion

*Rickettsia conorii *subsp*. israelensis*, belongs to the *R. conorii* complex. It was first reported in 1974 in Israel and initially, its distribution appeared to be restricted only to that country [6, 7]. *R. conorii *subsp*. israelensis* was first reported in Italy in 2005, in fact, it was identified in blood samples of patients with MSF collected from 1987 and 2001[8]. Other countries in which the microorganism is known to be present are Portugal, Israel, and other nearby Countries between the Maghreb and the Middle East [9].

ISF is a disease like MSF but characterized by higher severity. The tache noire in ISF was classically considered absent according to the first descriptions [10]. Conversely, De Sousa et al. in Portugal, reported a cohort in which the eschar was present in 17/45 patients [11]. Similarly, tache noire was present in 5/8 of the cases of ISF reported in Italy. In the study of De Sousa, gastrointestinal symptoms were a predictor of poor outcome; however, the pathophysiology of the gastrointestinal symptoms remained unclear. The study suggested that vomiting, nausea and diarrhea could be related to the rise in intracranial pressure and, therefore, to neurological involvement, or a consequence of the massive release of inflammatory cytokines. In ISF the injury to vascular endothelium is multi-systemic and more severe than in MSF. The manifestations can include confusion, for the involvement of the brain endothelium, tachypnea, and interstitial pneumonia (for the involvement of the lung endothelium and the sepsis status), and petechial exanthema (for the involvement of skin endothelium, thrombocytopenia and alterations of blood coagulation) [10]. Also, acute kidney injury due to sepsis and systemic hypoperfusion can occur. Some cases of purpura fulminans associated with ISF were reported in Israel [12, 13]. Imported fatal cases of ISF have also been described in the United Kingdom, in a traveler who returned from south Portugal and died in 2005 [14], and in a Swiss man after a cruise in the south Mediterranean Sea [15].

sHLH is a potentially fatal hyperinflammatory syndrome that is characterized by histiocyte proliferation and hemophagocytosis. It can be triggered by rickettsial diseases although this syndrome has never been associated with *R. conorii *subsp*. israelensis* [16]. We cannot exclude that in our case sHLH was present. In fact, the patient did not consent for bone marrow biopsy; however, the steroid treatment was followed by a rapid improvement in the clinical condition.

Interstitial pneumonia has been occasionally described in course of *rickettsial* infection especially in scrub typhus [17, 18]. In the Korean study of Song SW et al. scrub typhus patients with interstitial pneumonia (*n* = 52) had higher incidences in episode of hypoxia (*p* = 0.030), hypotension (*p* = 0.024), severe thrombocytopenia (*p* = 0.036) and hypoalbuminemia (*p* = 0.013) than the patients without interstitial pneumonia (*n* = 49). The patients with interstitial pneumonia also had higher incidences of pleural effusion (*p* < 0.001), focal atelectasis (*p* = 0.019), cardiomegaly (*p* < 0.001), pulmonary alveolar edema (*p* = 0.011) and hilar lymphadenopathy (*p* < 0.001) than the patients without interstitial pneumonia [19].

Pleural effusion has been rarely described in course of MSF [20]. Tarantino et al. in USA were able to show by microscopy morulae in polymorphonuclear leukocytes from pleural fluid in a case of ehrichiosis [21]. However, *Rickettsiales* had never been identified by culture or molecular methods in pleural effusion prior to our description.

In conclusion, ISF may be associated with interstitial pneumonia and pleural effusion. Their presence should not exclude a diagnosis of rickettsiosis, but they do indicate a greater severity and should not lead the physician away from this diagnosis.
